# Power Losses Models for Magnetic Cores: A Review

**DOI:** 10.3390/mi13030418

**Published:** 2022-03-07

**Authors:** Daniela Rodriguez-Sotelo, Martin A. Rodriguez-Licea, Ismael Araujo-Vargas, Juan Prado-Olivarez, Alejandro-Israel Barranco-Gutiérrez, Francisco J. Perez-Pinal

**Affiliations:** 1 Tecnológico Nacional de México, Instituto Tecnológico de Celaya, Antonio García Cubas Pte. 600, Celaya 38010, Mexico; d1903002@itcelaya.edu.mx (D.R.-S.); juan.prado@itcelaya.edu.mx (J.P.-O.); israel.barranco@itcelaya.edu.mx (A.-I.B.-G.); 2 CONACYT, Tecnológico Nacional de México, Instituto Tecnológico de Celaya, Antonio García Cubas Pte. 600, Celaya 38010, Mexico; martin.rodriguez@itcelaya.edu.mx; 3 Escuela Superior de Ingeniería Mecánica y Eléctrica (ESIME), Unidad Culhuacán, Instituto Politécnico Nacional, Mexico City 04260, Mexico; iaraujo@ipn.mx

**Keywords:** core losses methods, power losses, ferromagnetic material, inductors, transformers

## Abstract

In power electronics, magnetic components are fundamental, and, unfortunately, represent one of the greatest challenges for designers because they are some of the components that lead the opposition to miniaturization and the main source of losses (both electrical and thermal). The use of ferromagnetic materials as substitutes for ferrite, in the core of magnetic components, has been proposed as a solution to this problem, and with them, a new perspective and methodology in the calculation of power losses open the way to new design proposals and challenges to overcome. Achieving a core losses model that combines all the parameters (electric, magnetic, thermal) needed in power electronic applications is a challenge. The main objective of this work is to position the reader in state-of-the-art for core losses models. This last provides, in one source, tools and techniques to develop magnetic solutions towards miniaturization applications. Details about new proposals, materials used, design steps, software tools, and miniaturization examples are provided.

## 1. Introduction

It is a fact that magnetic components are an essential part of our lives. We can find them in almost anything of quotidian use, from simple things such as cell phone charges [1] to TVs and home appliances. However, they have become more relevant in the development of electric and hybrid vehicles, electric machines [2], renewable energy systems [3,4,5,6], and recently in implanted electronics [7,8] to open the possibility to micro-scale neural interfaces [9]. Inside these devices and systems, a power stage is conformed by magnetic and electronic components.

A few years ago, these power stages used to be bigger and heavier, and they had considerable energy losses making them less efficient. Nowadays, silicon carbide (SiC) and gallium nitride (GaN) switching devices have improved power electronics, making them smaller and faster [10,11]. Notwithstanding, magnetic components oppose miniaturization; these remain stubbornly large and lossy [12]. In a modern power converter, magnetics are approximately half of the volume and weight, and they are the main source of power losses [13,14].

The trend in power electronics has two explicit purposes for the design of magnetic components. The former is to make maximum use of magnetics capabilities, achieving multiple functions from a single component [12]. The latter consists of minimizing the size of magnetic components substituting ferrite (the leading material in their fabrication) with ferromagnetic materials [15]. Those materials have a higher saturation point than ferrite, high permeability and are based on iron (Fe) and metallic elements (Si, Ni, Cr, and Co). Some examples of these kinds of material are Fe-Si alloys, powder cores, amorphous materials, and nanocrystal material [16,17,18].

The design of magnetic components is the key to achieving two purposes. The design parameters are intimately dependent on geometric structure, excitation conditions, and magnetic properties such as power losses that determine if a core magnetic is suitable to be part of a magnetic component [19]. Power losses in magnetic components are important design parameters; which limit many high-frequency designs.

Power losses in a magnetic component are divided into two core power losses and winding power losses. Core power losses are related to the selection of the material to make the core of inductors and transformers; in this case, parameters such as frequency operating range, geometric shape, volume, weight, temperature operating range, magnetic saturation point, and relative permeability must be considered [20]. On the other hand, winding losses are related to phenomena such as skin effect, direct current (DC) and alternating current(AC) resistance in a conductor, and proximity effect [19,21,22]. Those phenomena increase the volume of a winding structure [23]. One way to overcome this kind of loss is by using Litz wire [24].

Nowadays, there are several models for studying, predicting, and analyzing the power losses in the ferromagnetic cores of magnetic components. However, many of them have been developed to work in a limited frequency range, temperature and low magnetic flux [25]. Usually, the ferromagnetic cores’ manufacturers provide the graphs of the core losses in their datasheets, which it is commonly obtained with sinusoidal signal tests and for specific values of frequency (*f*) and magnetic flux (*B*) [26].

There are many ways to calculate the power losses in magnetic materials. The original Steinmetz equation is one of them; however, to the best of the author’s knowledge, it is not always the best option. In the author’s opinion, selecting a method to calculate power core losses must be based on its versatility to vary their measurement’s parameters [27].

This document aims to provide the reader with a general panorama about the core power losses in ferromagnetic materials, emphasising the diverse models found in the literature, announcing their characteristics, advantages, and limitations. Besides, it will present a relationship about magnetic materials and tested models. Any magnetic component designer must know the basic methods, core losses’ models, the conditions at which they are valid, and their mathematical fundamentals. Magnetic and thermal losses, calculus and modelling are open research areas due to the non-linear features of ferromagnetic materials, the complexity of developing a unique power core losses model for the overall ferromagnetic materials, and their respective validations.

The organization of this work consists of the following sections. In Section 2, the reader will find information about the characteristics of each ferromagnetic material. Losses in magnetic components are reviewed in Section 3. In Section 4, general core losses models are mentioned as empirical core losses proposals. Features of Finite Element Method software are given in Section 5. The design process of a magnetic component is discussed in Section 6; on the other hand, the importance of the core losses methods in miniaturization of magnetic devices is summarized in Section 7. The discussion of this work is presented in Section 8, and finally, in Section 9, conclusions are provided.

## 2. Ferromagnetic Alloys

Ferromagnetic materials are exciting materials, where several of their physical properties and chemical micro-structure allow being controlled. Susceptibilities, permeabilities, the shape of the hysteresis loop, power loss, coercivity, remanence, and magnetic induction are some examples of no intrinsic properties [18]. The saturation magnetization and the Curie temperature are the only intrinsic properties [17,18].

The magnetic behavior is ruled by the dipole moments’ interaction of their atoms within an external magnetic field [28]. Ferromagnetic materials have strong magnetic properties due to their magnetic moments that tend to line up easily along an outward magnetic field direction [29]. These materials also have the property of remaining partially magnetized even when the external magnetic field is removed; this means that they can quickly change their magnetic polarization by applying a small field. Ferromagnetic materials are also profitable materials due to being composed by Fe, one of the eight more abundant elements on Earth [30].

**Figure 1:**
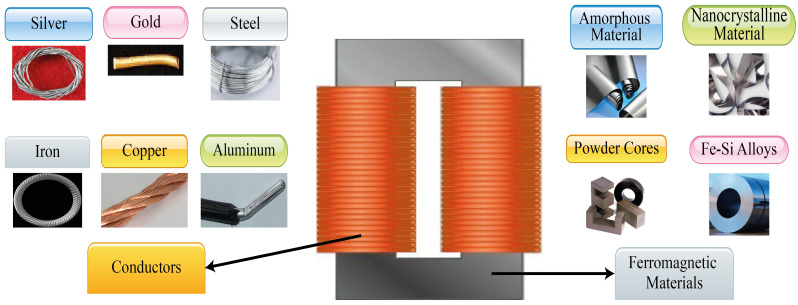
Ferromagnetic and conductor materials types. Source: Adapted from [33,34,35,36,37,38,39,40,41,42,43].

Ferromagnetic materials are used in the core of magnetic components; they are classified in Fe-Si alloys, powder cores, amorphous material, and nanocrystalline material; on the other hand, materials as silver, gold, copper, aluminium, iron, steel, among others, are used in windings Figure 1. Except for powder cores, the rest of them are rolled materials [31]; in the following sections, the importance of this fact in the core losses calculation will be detailed. Each one has specific magnetic properties, manufacturing processes, and physical features that determine its feasibility [32].

Fe-Si alloys are alloys based on Fe with small quantities of Si (not more than 4.5%). These alloys are attractive due to their reasonable cost and magnetic properties [44]. There are two kinds: grain non-oriented sheets (GNO) and grain-oriented sheets (GO) [30]. At present, GO material represents 80% of the electrical devices market [17,45].

On the other hand, powder cores are fabricated from metallic powders, typically iron; however, those can be composed of alloys with P, Si and Co [46]. The manufacturing process of this kind of material allows special fabrication geometry cores. Usually, powder cores are mixed with a binder or insulating material to reduce magnetic losses at high frequencies [47]. According to the material used in their fabrication, those can be classified into four groups: iron powder core, molybdenum permalloy powder cores (MPP), high flux powder cores, and sendust cores, also called Kool Mμ [48]. These materials present the more recent advances in magnetic elements [49].

Amorphous alloys or metallic glasses are materials without crystalline order [50]. Those alloys are very strong and hard, but also ductile. They contain approximately 80% of particles of Fe, Ni, Co, and their combinations; and 20% of metalloids particles or glass formed elements (C, Al, B, Si, and P) [51,52].

Nanocrystalline alloys consist of a Fe-Si ultra-thin grain alloy with a few quantities of Cu and Nb. Their manufacturing process is very similar to amorphous alloys [53]. Nanocrystals are materials with high mechanical hardness and extremely fragile. The four main kinds are: finemet, nanoperm, hitperm, and nanomet [54,55].

Nanocrystalline, amorphous and Fe-Si alloys are the materials more used in power electronics, so they have been widely studied, especially in terms of core power losses. In rolled materials as Fe-Si alloys and amorphous materials, their magnetic properties depend on the sheet’s thickness (around a few mm, and 5–50 μm, respectively). Instead, in nanocrystalline alloys, magnetic properties depend on the diameter of their grains (of the order of 10–15 nm). Rolled materials are susceptible to Eddy currents and skin losses. In contrast, powder cores’ magnetic properties can be manipulated during their manufacturing process, which includes the relative permeability variation according to the magnetic field intensity, high saturation point, fringing flux elimination, soft saturation, among others.

## 3. Losses in Magnetic Components

To any magnetic component designer, a real challenge to overcome is getting a magnetic component with high efficiency, small size, low cost, convenience, and low losses [56]. Usually, losses are the common factor in all requirements announced before; losses are the most difficult challenges to beat in a magnetic component.

**Figure 2:**
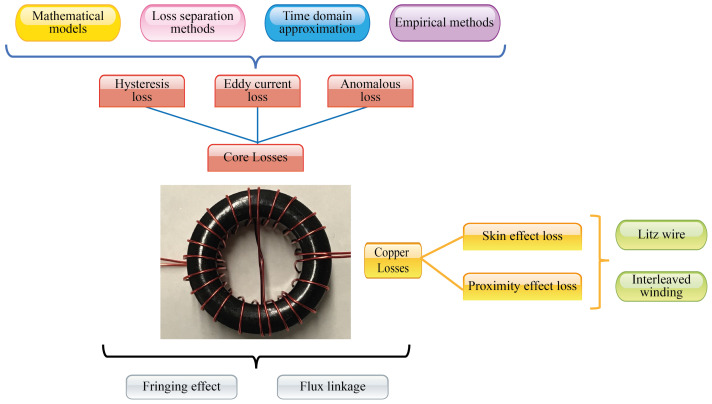
Types and classification of losses presented on magnetic components. Source: Adapted from [58].

Losses in a magnetic component are divided in two groups: core losses and winding losses (also called copper losses), [57]. Figure 2 shows each one of them, as well as their causing phenomena, methods, models, techniques and elements associated with them; nonetheless, phenomena such as the fringing effect and the flux linkage many times are not considered in the losses model. Still, they are necessary to achieve a complete losses model in any magnetic component.

Current windings generate the flux linkage density; therefore, it is the sum of the flux enclosed of each one of the turns wound around the core, the flux linkage linked them [50,59]. Flux linkage depends on the conductor’s geometry and the quantity of flux contained in it. In the gap between windings is the maximum flux linkage [60,61,62].

Otherwise, the fringing effect is the counterpart of flux linkage; this is, the fringing effect is presented around the air gap instead of the windings of the magnetic component. This phenomenon depends on core geometry and core permeability, to higher permeability the fringing effect is low [50].

The copper losses are caused by the flow of DC and AC through the windings of a magnetic component, where losses always are more significant for AC than DC [63,64]. The circulating current in the windings generates Eddy phenomena as Eddy currents, skin effect loss and, proximity effect loss.

The skin effect and the proximity effect loss are linked to the conductor size, frequency, permeability and distance between winding wires. For the first of them, the current distribution trough the cross-area of the wire will define the current density in it. The skin effect and the proximity effect loss are linked to the winding wires. For the first of them, the current distribution through the cross-area of the wire will define the current density in it. It means that the conductor will have a uniform distribution for DC, higher on its surface and lower in its center for AC distribution [65].

The proximity effect is similar to the skin effect, but in this case, it is generated by the current carried nearby conductors [50,65]. Eddy currents are induced in a wire in this kind of loss due to a variant magnetic field in the vicinity of conductors at high frequency [46,66].

The Litz wire and interleaved windings help minimize winding losses in magnetic components, both are widely used currently. Indeed, interleaved windings are very efficient in high-frequency planar magnetics [67,68].

On the other hand, core losses directly depend on the intrinsic and extrinsic core materials’ characteristics. Core losses are related to hysteresis loop, Eddy currents, and anomalous or residual losses. Without matter, the core losses model chosen by the magnetic component designer will be based on those three primary losses; core loss models will be detailed in the next section. Besides, core losses depend on the core’s geometry and the core intrinsic properties’ such as permeability, flux density, Curie temperature, among others [69,70,71]. Many methods and models have been developed; all of them have the predominant interest in studying, analyzing and understanding those kinds of losses to improve the magnetic components’ performance.

## 4. General Core Losses Models

In a magnetic component, the core is the key to determine its magnetic properties and performance [72]. So to achieve an optimal magnetic component’s performance, the core losses effects must be characterized [73].

Core loss depends on many aspects that must be considered [74,75]:

- Relative permeability.
- Magnetic saturation point.
- Temperature operation range.
- AC excitation frequency and amplitude.
- Voltages’ waveform.
- DC bias.
- Magnetization process.
- Peak-to-peak value of magnetic flux density.

In the case of the magnetization process, some factors are instantaneous values and time variation values. While to waveform’s topic, the duty ratio of the excitation waveform also influence the core loss [76,77].

Generally, the core loss is provided at a specific frequency and a maximum flux density [78]. The variation frequency effect in ferromagnetic materials is related to Eddy currents and the wall-domain displacement [72].

Core losses in ferrimagnetic and ferromagnetic materials are similar. Both have losses due to Eddy currents, hysteresis, and anomalous; however, there are differences concerning flux density, magnetization process, and hysteresis loop shape that define the magnetic behavior of each one.

The hysteresis is one of the principal features of ferromagnetic materials; it describes the internal magnetization of magnetic components as a function of external magnetizing force and magnetization history [79]. The source of hysteresis loss is the domain wall movement and the magnetic domains’ reorientations [80,81].

The hysteresis loss is defined as power loss in each cycle of magnetization and demagnetization into a ferromagnetic material [82]. If a magnetic sample is excited from zero to the maximum field value and later comes back at the initial field’s value, it will be observed that the power returned is lower than the supplied it [83,84].

The loss is proportional to the area surrounded by the upper and lower traces of the hysteresis curve; it represents the per cycle loss and it is proportional to $f\cdot B^{2}$ [82]. However, if the curve’s shape remains equal for each successive excitation, the loss power will be the product of the core’s area and the applied frequency [80,82].

The hysteresis loops give a lot of information about the magnetic properties [72]. An accurate way to calculate core loss is by measuring the full hysteresis curve [85].

**Figure 3:**
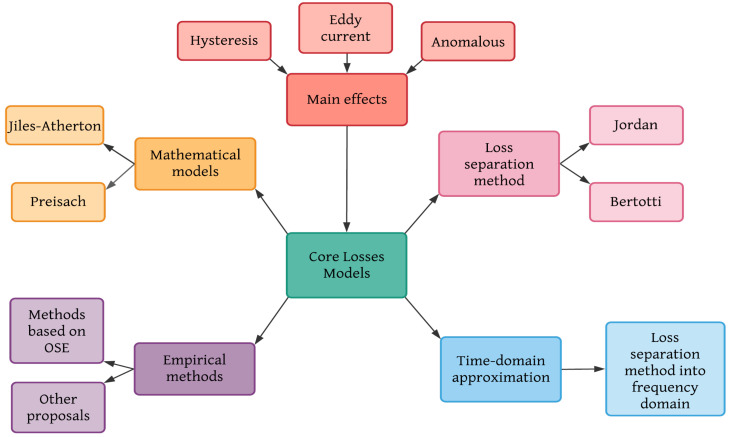
Classification of core losses methods.

There are many methods to calculate core losses; Figure 3 shows a general classification. All of them are based on one, two, or three main effects (hysteresis, Eddy current, and anomalous); depending on the method’s focus they are analyzed as macro or micro phenomena. Each one of the methods shown in Figure 3 will be detailed in the following paragraphs.

### 4.1. Mathematical Models

Mathematical hysteresis modelling is divided into approaches based on the theory of micro-magnetics and the methods based on curve-fitting. The hysteresis models require complex computation to calculate the model parameters, the material parameter that manufacturers do not provide, or mathematical approximations where the accuracy depends on the number of data points to fit hysteresis loops [86,87]. These drawbacks remain if they are analyzed from a purely mathematical or a physical point of view [78].

The Preisach model and the Jiles-Atherton (J-A) model have been widely used in practical problems to calculate core loss, they are in continuous improvement, and they are considered a valuable and convenient tool to the hysteresis modelling [87,88,89].

The Preisach is a scalar-static model that considers several quantities of basic’s domain-walls [72]. It is an accurate-phenomenological model, such that it could describe any system that shows a hysterical behavior [87,90]. This model can be a link between theory and experimentation to describe a microscopical system by measuring macroscopic behavior [78].

**Figure 4:**
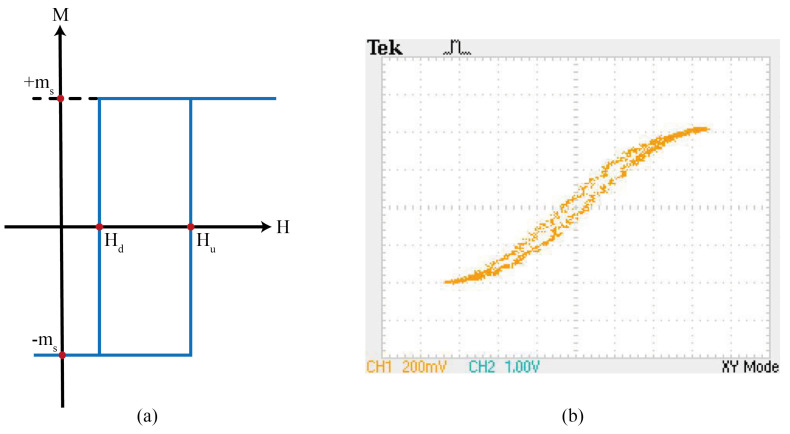
Hysteresis loop of (**a**) an oriented magnetic domain, (**b**) a multidomain particle of a 3C90 Epcos ferrite core at 500 Hz and 28.5 ${}^{\circ }$C. Source: Adapted from [91].

Given a typical hysteresis loop for separate domains as shown in Figure 4, $H_{d}$ and $H_{u}$ are the switching magnetic fields “down” and “up”, respectively. The magnetization M(t) of a particle having the hysteresis loop $\hat{m}\left(H_{u},H_{d}\right)$ is described as the magnetic moment $m_{s}$ while the particle is switched up $\hat{m}\left(H_{u},H_{d}\right)H\left(t\right)=+m_{s}$ or down $\hat{m}\left(H_{u},H_{d}\right)H\left(t\right)=-m_{s}$ [87]. It is assumed that all domains have a distribution of reversal fields $H_{u}$ and $H_{d}$ that can be characterized by a distribution function $\phi (H_{u},H_{d})$, so the Preisach model is usually defined as:

(1) $$M\left(t\right)={\;\int \int \;}_{H_{u}\geq H_{d}}\phi \left(H_{u},H_{d}\right)*\hat{m}\left(H_{u},H_{d}\right)*H\left(t\right)dH_{u}dH_{d}.$$

A drawback of the Preisach model is the several measurement data to adapt it to the B-H environment’s changes [72]. Nonetheless, it can track complex magnetization processes and minor loops [78].

On the other hand, the J-A model requires solving a strongly non-linear system of equations, with five scalar parameters, which are determined in an experimental way; this is, measuring the hysteresis loop [92]. This model predicts the major hysteresis loops under quasi-static conditions, besides there is a dynamic conditions version in which eddy current loss, DC-bias fields, anisotropy, and minor loops are included [86,93].

The J-A model is described as the sum of reversible $M_{rev}$ and irreversible $M_{irr}$ magnetizations, hence, the total magnetization *M* is:

(2) $$M=M_{irr}+M_{rev}$$

(3) $$\frac{dM_{rev}}{dH}=c\left(\frac{dM_{an}}{dH}-\frac{dM_{irr}}{dH}\right)$$

(4) $$\frac{dM_{irr}}{dH}=\frac{M_{an}-M_{irr}}{\delta x/\mu_{0}-\alpha_{ic}\left(M_{an}-M_{irr}\right)}$$

(5) $$M_{an}=M_{s}\left(\coth\frac{H_{eff}}{a_{s}}-\frac{a_{s}}{H_{eff}}\right)$$

(6) $$H_{eff}=H+\alpha_{ic}M.$$

In these equations, *c* is the reversibility coefficient, $\alpha_{ic}$ is the inter-domain coupling, δ indicates the direction for magnetizing field (δ=1 for the increasing field, and δ=−1 for decreasing field), *x* is the Steinmetz loss coefficient, $M_{s}$ is the saturation magnetization, $M_{an}$ is anhysteretic magnetization, $H_{eff}$ is the effective field, *H* is the magnetic field, $\mu_{0}$ is the permeability in free-space, and $a_{s}$ is the shape parameter for anhysteretic magnetization [73]. A drawback of the J-A model is the computing time and the computing resources to solve a strongly non-linear system.

On the other hand, Eddy’s losses are produced due to changing magnetic fields inside the analyzed core. These fast changes generate circulating peak currents into it, in the form of loops, generating losses measured in joules [94].

These losses depend on the actual signal shape instead of only the maximum value of the density flux. The power loss is proportional to the area of the measured loops and inversely proportional to the resistivity of the core material [50,94].

Eddy currents are perpendicular to the magnetic field axis and domain at high-frequency, as well, they are proportional to the frequency. This phenomenon can be treated as a three-dimensional character or in a simplified way [95]. In a magnetic circuit, Eddy currents cause flux density changes in specific points of its cross-section [96].

One way to describe the Eddy currents distribution is based on Maxwell’s equations [95,97]. In practice, especially on higher frequencies, there is a difference in the measured total loss between the sum of the hysteresis loss and the Eddy current loss; this difference is known as anomalous or residual losses [13]. The effect of temperature and the relaxation phenomena are enclosed in this category [82].

### 4.2. Time-Domain Approximation Models

For the time-domain model, the loss separation method is used in the frequency domain, using Fast Fourier Transform (FFT) [13].

Time-domain approximation (TDA) can be used for sinusoidal and non-sinusoidal magnetic flux density; however, it is only valid for linear systems [98]. The losses are calculated considering each frequency, separately, and adding them later [85,98].

(7) $$P_{c}=\sum_{n=1}^{\infty }\pi \left(fn\right)B_{n}H_{n}\sin\phi_{n}.$$

According to Equation (7), time-domain approximation is a function of the fundamental frequency *f*, the peak values of the *n*th harmonic of the magnetic field *H* and magnetic flux density *B*, and $\phi_{n}$ the angle between $B_{n}$ and $H_{n}$.

When a pulse width modulation (PWM) is induced in a magnetic component made with non-linear material, a non-sinusoidal ripple is generated [93]. Fourier’s core loss decomposition accuracy is not acceptable for non-sinusoidal flux densities and it is limited for frequencies > 400 Hz, as it was reported in [99].

### 4.3. Loss Separation Models

The loss separation method (LSM) is well-known, accurate and straightforward in many applications [94]. It defines the core loss ($P_{C}$) under a dynamic magnetic excitation as the sum of three components: the hysteresis loss ($P_{h}$), the Eddy current loss ($P_{eddy}$) and the residual or anomalous loss ($P_{anom}$) [82] is given by:

(8) $$P_{c}=P_{h}+P_{eddy}+P_{anom}.$$

According to Equation (8), the power loss per unit volume is the sum of a hysteresis and dynamic contribution; Eddy currents and residual loss are part of the latest one [100].

The loss separation method was proposed in 1924 by Jordan, which describes the core losses as the sum of the hysteresis loss (or static loss) and the Eddy loss (or dynamic loss) [98]

(9) $$P_{c}=k_{h}fB_{s}+k_{eddy}f^{2}B_{s}^{2}.$$

Years later, Bertotti extended Jordan’s proposal, adding an extra term to calculate residual losses. G. Bertotti is the maximum exponent in the loss separation methods; his theory provides a solid physical background. The total power loss can be calculated at any magnetizing frequency as the sum of three components [44]. This is show in the following equation

(10) $$P_{c}=k_{h}fB_{s}^{x}+k_{eddy}f^{2}B_{s}^{2}+k_{anom}f^{1.5}B_{s}^{1.5},$$

where $k_{h}$, $k_{c}$, $k_{anom}$ are the hysteresis, Eddy currents, and anomalous coefficients, respectively. The hysteresis coefficient decreases if the magnetic permeability increase. The frequency is represented by *f*, while *x* is the Steinmetz coefficient and has values between 1.5 and 2.5, according to the permeability of the material. Finally, $B_{s}$ is the peak value of the flux density amplitude [23,100].

From Equation (10), Bertotti defined eddy currents and anomalous losses in Equations (11) and (12), respectively, for lamination materials.

(11) $$P_{eddy}=\frac{\pi^{2}d^{2}f^{2}B_{s}^{2}}{\rho \beta }$$

(12) $$P_{anom}=8\sqrt{\frac{GAV_{0}}{\rho }}B_{s}^{1.5}\sqrt{f},$$

where *G* is about 0.2, ρ is the electrical resistivity, *d* is the lamination thickness, *A* is the cross sectional area of the lamination, β is the magnetic induction exponent, *G* is a dimensionless coefficient of Eddy current, and $V_{0}$ is a parameter that characterizes the statistical distribution of the magnetic objects responsible for the anomalous eddy currents [101]. However, this method only provides average information, so it is not able to calculate core loss under harmonic excitation [94]. Still, it can calculate core loss under square waveforms considering DC bias [102].

### 4.4. Empirical Models

A big group of empirical models is based on a simple power-law equation, which was proposed in 1982 by Charles Steinmetz to calculate hysteresis loss without including the frequency relation [103]. This equation is known as the Steinmetz equation or original Steinmetz equation (OSE),

(13) $$P_{\mathrm{OSE}}=kf^{\alpha }B_{s}^{\beta },$$

where $P_{\mathrm{OSE}}$ is the time-average core loss per unit volume, $B_{s}$ the peak induction of the sinusoidal excitation, *k* is a material parameter, α, and β are the frequency and magnetic induction exponents, respectively, often referred to as Steinmetz parameters [76]. Typically α is a number between 1 and 2, and β is typically between 1.5 and 3 [104].

The Steinmetz parameters can be determined from a double logarithm plot by linear curve fitting of the measured core loss data. Therefore Equation (13) assumes only sinusoidal flux densities with no DC bias [76,77,98,105].

In modern power electronics applications, sinusoidal-wave voltage excitation is not practical because many of them, like power converters, require square-wave voltage excitation [106]. Therefore, a square waveform’s core loss can be lower than the sinusoidal-wave’s losses for the same peak flux density and the same frequency [76,107].

To overcome the aforementioned situation, modifications to OSE have been made. The result was the developing Modified Steinmetz Equation(MSE) [108], General Steinmetz Equation (GSE) [109], Doubly Improved Steinmetz Equation (Improved-Improved Steinmetz Equation, *i*${}^{2}$GSE) [110], Natural Steinmetz Equation (NSE) [85], and Waveform-Coefficient Steinmetz Equation (WcSE) [111]. It is important to mention that the OSEs’s constants α, β, and *k* remain in those expressions.

Modified Steinmetz Equation was the first modification proposed to calculate core loss with non-sinusoidal excitation, incorporating into OSE the influence of a magnetization rate. MSE is proposed in [112] as follows

(14) $$P_{\mathrm{MSE}}=\left(kf_{eq}^{\alpha -1}B_{s}^{\beta }\right)f_{r}$$

$$f_{eq}=\frac{2}{\Delta B^{2}\pi^{2}}\int_{0}^{T}{\left(\frac{dB(t)}{dt}\right)}^{2}dt,$$

where $f_{r}$ is the periodic waveform fundamental frequency, $f_{eq}$ is the frequency equivalent, ΔB is the magnetic induction peak to peak, and dB(t)/dt is the core loss magnetization rate.

A drawback of Equation (14) is that its accuracy decreases with the increasing of waveform harmonics, and for waveforms with a small fundamental frequency part [77,85,113].

Another modification of OSE is the Generalized Steinmetz Equation to overcome the drawbacks of OSE and MSE for sinusoidal excitations, whose expression is:

(15) $$P_{GSE}=\frac{k_{1}}{T}\int_{0}^{T}{\left|\frac{dB(t)}{dt}\right|}^{\alpha }{\left|B(t)\right|}^{\beta -\alpha }dt$$

$$k_{1}=\frac{k}{2^{\beta -\alpha }{\left(2\pi \right)}^{\alpha -1}\int_{0}^{2\pi }{\left|\cos\theta \right|}^{\alpha }{\left|\sin\theta \right|}^{\left(\beta -\alpha \right)}d\theta }.$$

In this equation, the current value of flux is considered additional to the instantaneous value of dB(t)/dt, B(t), *T* is the waveform period, and θ is the sinusoidal waveform phase angle. The main GSE’s advantage is it DC-bias sensitivity. However, its accuracy is limited if a higher harmonic part of the flux density becomes significant [85,98].

The Improved Generalized Steinmetz Equation is considered one of the best methods because it is a practical and accurate [114]. It is defined as follows:

(16) $$P_{i\mathrm{GSE}}=\frac{k_{i}}{T}\int_{0}^{T}{\left|\frac{dB(t)}{dt}\right|}^{\alpha }{\left|\Delta B\right|}^{\beta -\alpha }dt$$

$$k_{i}=\frac{k}{2^{\beta -\alpha }{\left(2\pi \right)}^{\alpha -1}\int_{0}^{2\pi }{\left|\cos\theta \right|}^{\alpha }d\theta }.$$

Unlike GSE, which uses the instantaneous value B(t), *i*GSE considers its peak-to-peak value ΔB. Any excitation waveform can be calculated by it; therefore, it has better accuracy with waveforms that contained strong harmonics [114,115,116,117].

The Natural Steinmetz Extension is similar approach to the *i*GSE; this means that ΔB was also taken into account [85,118]. It is written as:

(17) $$P_{NSE}={\left(\frac{\Delta B}{2}\right)}^{\beta -\alpha }\frac{k_{N}}{T}\int_{0}^{T}{\left|\frac{dB(t)}{dt}\right|}^{\alpha }dt$$

$$k_{N}=\frac{k}{{\left(2\pi \right)}^{\alpha -1}\int_{0}^{2\pi }{\left|\cos\theta \right|}^{\alpha }d\theta }.$$

The NSE focuses on the impact of rectangular switching waveform like PWM, so Equation (17) can be modelled for a square waveform with duty ratio *D* by:

(18) $$P_{NSE}=k_{N}{\left(2f\right)}^{\alpha }B_{s}\left[D^{1-\alpha }+{\left(1-D\right)}^{1-\alpha }\right].$$

The Improved–Improved Generalized Steinmetz Equation (*i*${}^{2}$GSE) considers the relaxation phenomena effect in the magnetic material used due to a transition to zero voltage [115]. The $i^{2}$GSE was developed to work with any waveform but its main application is with trapezoidal magnetic flux waveform [110]. For a trapezoidal waveform, *i*${}^{2}$GSE is described as follows:

(19) $$P_{i^{2}\mathrm{GSE}}=\frac{1}{T}\int_{0}^{T}k_{i}{\left|\frac{dB(t)}{dt}\right|}^{\alpha }{\left(\Delta B\right)}^{\left(\beta -\alpha \right)}dt+\sum_{l=1}^{n}Q_{rl}P_{rl}$$

$$P_{rl}=\frac{k_{r}}{T}{\left|\frac{d}{dt}B\left(t-\right)\right|}^{\alpha_{r}}{\left(\Delta B\right)}^{\beta_{r}}\left(1-e^{-\frac{t_{1}}{\tau }}\right)$$

$$Q_{rl}=e^{-q_{r}\left|\frac{dB(t+)/dt}{dB(t-)/dt}\right|}.$$

Note that $P_{rl}$ and $Q_{rl}$ calculate the variation of each voltage change and the voltage change, respectively. However, to use the $i^{2}$GSE requires additional coefficients as can be seen in Equation (19) where $k_{r}$, $\alpha_{r}$, $\beta_{r}$, τ and $q_{r}$ are material parameters and they have to be measured experimentally; given that, they are not provided by manufacturers and the steps to extract the model parameters are detailed in [119]. The expression given by Equation (19) can also be rewritten by a triangular waveform.

Finally, the Waveform-Coefficient Steinmetz Equation (WcSE) was proposed in [111] to include the resonant phenomena, and it applies only at situations with certain loss characteristics. This empirical equation is used in high-power, and high-frequency applications, where resonant operations are adopted to reduce switching losses. The WcSE is a simple method that correlates a non-sinusoidal wave with a sinusoidal one with the same peak flux density; the waveform coefficient (FWC) is the ratio between the average value of both types of signals [119,120]. WcSE can be written as follows:

(20) $$P_{\mathrm{WcSE}}=FWCkf^{\alpha }B_{s}^{\beta }.$$

The most important characteristics of each approximation based on the Steinmetz equation are listed in Table 1.

In addition to the methods listed before, in [110] the authors provided several graphs for different materials at different operating temperatures making use of the Steinmetz Premagnetization Graph (SPG), which is a simple form to show the dependency of Steinmetz’s parameters on premagnetization to calculate the core losses under bias conditions.

Using the SPG, the changes of Steinmetz in *i*GSE can be considered; it is also possible to calculate the core loss under any density flux waveform [19,74].

#### Empirical Core Losses Proposals

The primary purpose of this section is to show a few empirical core losses models proposals to emphasize the diversity of parameters that are taken into account: voltage waveforms, temperature effect, and the versatility of using them to calculate core losses.

Generally, square waveforms are used in power electronic applications resulting in triangular waveform induction and flux density; therefore, core losses models must contemplate this waveform. However, a sinusoidal approximation for a duty rate of 50 % is valid [121].

Curve fitting is used to approximate and characterize the model’s core losses parameters. Nonetheless, curve fitting by polynomials are unstable with minor changes at entry, resulting in a big variation on coefficients, so the designer must be meticulous in selecting the model as the curve fitting method and logarithm curve [121].

The Composite Waveform Hypothesis (CHW) was proposed in 2010; it is based on a hypothesis that establishes that if a rectangular waveform given is decomposed in two pulses, total core losses are the sum of the losses generated by each one of the two pulses [26,122]. This method is described by

(21) $$P_{\mathrm{CHW}}=\frac{1}{T}\left[P_{sqr}\left(\frac{V_{1}}{N},t\right)t_{1}+P_{sqr}\left(\frac{V_{2}}{N},t\right)t_{2}\right],$$

where $P_{sqr}$ is the power losses of a rectangular waveform with the parameters given, $V_{1}/N$ and $V_{2}/N$ are the voltage by turn in time $t_{1}$ and $t_{2}$, respectively.

Villar’s proposal [120,123] calculates core losses for three-level voltage profiles through a lineal model by parts. Villar takes the models based on Steinmetz equations and modifies them by a linear model by parts (22), (23) and (24); this includes a duty ratio parameter in the original equations, getting expressions to calculate core losses for three-level voltage profiles.

(22) $$P_{Villar\mathrm{OSE}}=kf^{\alpha }B_{s}^{\beta }D^{\beta }$$

(23) $$P_{VillarI\mathrm{GSE}}=2^{\alpha +\beta }k_{i}f^{\alpha }B_{s}^{\beta }D^{\beta -\alpha +1}$$

(24) $$P_{Villar\mathrm{WcSE}}=\frac{\pi }{4}\left(1+\frac{\omega }{\pi }\right)kf^{\alpha }B_{s}^{\beta }D^{\beta }.$$

In Equation (24) the parameter ω is the duration of zero-voltage period this is, power electronics converters’ switching devices take a short time to start their conduction mode; therefore, Villar includes this effect in its model by the flux density’s equation as follows:

(25) $$B_{s}=\frac{1}{2}\frac{U}{NA_{eff}}\left(\frac{T}{2}-T_{\omega }\right),T_{\omega }=\omega \frac{T}{2\pi },$$

where *U* is a DC constant voltage, $A_{eff}$ is the core’s area effective, and $T_{\omega }$ is the length of zero-voltage period.

Villar’s proposal also is applied to the equivalent elliptical loop (EEL); however, the proposal does not include thermal parameters.

Another alternative is the model proposed by Gorécki given by Equation (26) in [124,125], which includes 20 parameters. Those are divided into three groups: electrical parameters, magnetic parameters, and thermal parameters. At the same time, the magnetic parameters’ are subdivided into three groups: core material parameters, geometrical parameters, and ferromagnetic material parameters corresponding to power losses. The model parameters can be obtained by the datasheets of the cores and experimental way when the inductor is tested under specific conditions, which is called local estimation.

(26) $$P_{Gorecki}=P_{v0}f^{\alpha }B_{s}^{\beta }{\left(2\pi \right)}^{\alpha }\left[1+\alpha_{p}{\left(T_{R}-T_{M}\right)}^{2}\right]\left[0.6336-0.1892\ln\left(\alpha \right)\right]$$

$$P_{v0}=\alpha exp\left(-\frac{f+f_{0}}{f_{3}}\right)+a_{1}\left(T_{R}-T_{M}\right)+a_{2}exp\left(\frac{f-f_{2}}{f_{1}}\right)$$

$$\beta \left\{\begin{array}{ccc} 2\left[1-exp\left(-\frac{T_{R}}{\alpha_{T}}\right)\right]+1.5 & \;\mathrm{if} & exp\left(\frac{-T_{R}}{\alpha_{T}}\right)>0\\ 1.5 & \;\mathrm{if} & exp\left(\frac{-T_{R}}{\alpha_{T}}\right)<0 \end{array}.\right.$$

In these equations, $T_{R}$ is the core temperature, $T_{M}$ is the core temperature at which the core has a minor loss, $\alpha_{p}$ is the losses’ temperature coefficient in ferromagnetic material, *a*, $a_{1}$, $a_{2}$, $f_{0}$, $f_{1}$, $f_{2}$, $f_{3}$, and $\alpha_{T}$ are material parameters.

Gorecki’s model is also valid for the triangular waveform following Equation (27), where *D* is the duty ratio of the waveform.

(27) $$P_{Gorecki}=P_{v0}f^{\alpha }B_{s}^{\beta }{\left(2\pi \right)}^{\alpha }\left[1+\alpha_{p}{\left(T_{R}-T_{M}\right)}^{2}\right]\left[D^{\left(1-\alpha \right)}+{\left(1+D\right)}^{\left(1-\alpha \right)}\right].$$

The main drawback of Gorecki’s model is to find the overall parameters mentioned before, and solving the equations related to the power core losses.

## 5. Simulation Software

Several core loss models have been reviewed; they are the base for developing a series of numerical and theoretical models that are useful for designing magnetic components. Nonetheless, they fail to predict dynamic magnetic behaviors. Usually, the calculation of the core parameters’ losses in a dynamic situation is complicated (specially with complex geometries), and it requires a rigorous numerical treatment [126].

Several methods are used in the simulation and calculation of core and wire windings. However, the finite element method (FEM) is the most widely used for designs in 2-D and 3-D [22].

In 1960 Clough introduced the name of Finite Element Method (FEM), which continues to these days [127]. FEM is a computational method whose basic idea consists on finding a complicated problem and replacing it with a simpler one. It is always possible to improve the approximation solution spending more computational effort [127,128].

To find the solution of a region, the FEM considers that it is built of many small, interconnected subregions called elements [128], and the global solution is obtained from the union of individual solutions on these regions [129].

The FEM is a tool for solving problems with partial differential equations that are part of physics problems [130]. FEM has quite benefits of using it; some of them is the freedom that it offers in the discretization’s selection, and its well developed theoretical base that allows valid error estimates for the numerical model equations, and its flexibility to be adapted to a wide range of numerical problems [129,131].

The FEM was originally developed to solve problems in solid-state mechanics. Still, its versatility, excellent simulation technique, availability to optimize the mesh size, and accuracy have been implemented in a wide variety of applied science and engineering [127,129].

Mathematical models are discretized by FEM, resulting in numerical models. To solve the discretized equations, Finite Element Analysis (FEA) is used [131,132].

There are many FEM software developers in the market; however, Ansys^®^ is the leader so far. Ansys, Inc. (Canonsburg, PA, USA) was founded in 1970s, and since then, it has developed, commercialized and brought support at several range of physics through engineering simulation software. The Ansys^®^ catalogue includes simulation software for semiconductors, structures, materials, fluids, and electronics.

**Figure 5:**
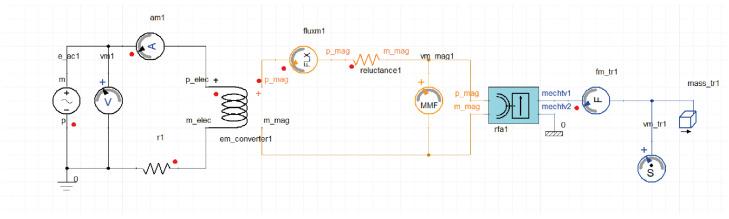
Twin Builder example.

An exciting tool that Ansys has developed in recent years is Twin Builder^®^, a multi-technology platform to create digital representations simulations, recollected real-time data information through sensor inputs asset with real-world [133]. Twin Builder^®^ is a powerful and robust multi-domain system modelling compatible with a series of standard languages and formats as SPICE, Python, C/C++, simplorer modelling language (SML), among others. It can be used to develop basic simulation experiments and advanced simulation studies, from 2-D and 3-D physics simulations. Inclusive the functional mock-up interface (FMI standard) can import and export models as available mockup units (FMU). An example of an electromagnetic digital twin is shown in Figure 5. Twin Builder^®^ only is compatible with Ansys software and its main application is in the industry as virtual laboratory to test any kind of system.

**Figure 6:**
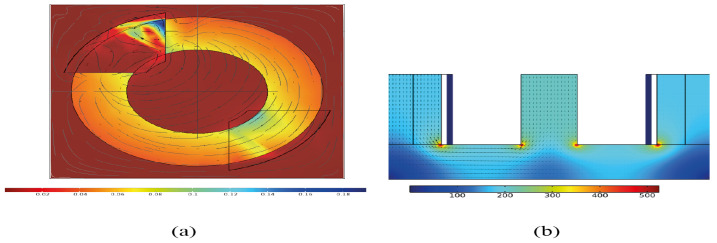
Examples of magnetic flux density simulations’ in Comsol Multiphysics for a powder core with shape: (**a**) toroidal, and (**b**) “E”.

Added to these FEM software developers, there is Comsol Multiphysics^®^, which is a powerful software tool for developing and simulating modelling designs in all fields of engineering, manufacturing, and scientific research [134]. This software was founded in 1986. Its main characteristic is its friendly graphical user interface. The Comsol Multiphysics^®^ community is more significant than the other software. In Figure 6, two simulations are provided. Comsol Multiphysics^®^ can import designs in CAD and export final designs to Simulink^®^, given that both belong to the MathWorks^®^ family. Comsol Multiphysics^®^ is oriented to students and academic researchers, it is not complex to learn and it is an excellent option to start with for FEM software.

Another big company of FEM software is JMAG^®^, a simulation software specialized for electric device design and development, including the accurate model of complex magnetic phenomena. It was founded in 1983, and currently, its most attractive feature is the capability to link various systems through its interface [135].

**Figure 7:**
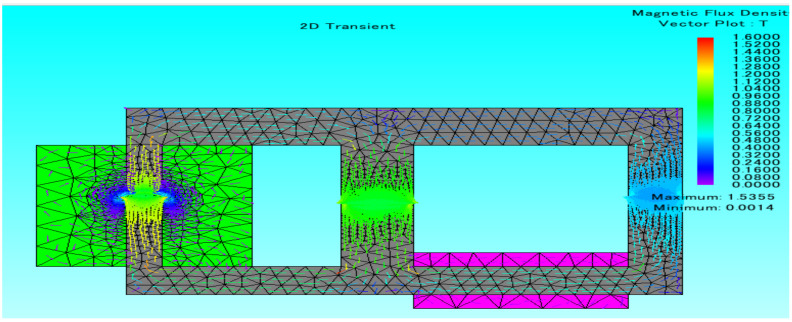
JMAG example of magnetic flux density simulation.

The JMAG^®^ interface allows the data exchange at high speed without loss of precision with other software as SPEED, PSIM and MATLAB/Simulink; one example of this software is shown in Figure 7. Additionally, JMAG^®^ allows to the user import and export multi-purpose files and to run VB Script and other scripting languages [135].

Another feature of JMAG^®^ is its CAD interface to link and import files with software as SOLIDWORKS, CATIA V5, among others to test the model developed using software in the loop (SIL), model in the loop (MIL) and hardware in the loop (HIL) systems, which are applied for system-level and real-time simulations [135,136]. JMAG^®^ is a specialized software to model inductors and magnetic motors adding vibration and thermal analysis; however, the information about it is limited, and it is complex to learn.

The software mentioned before are the most commonly used; however, there are many other finite element software packages available to any platform and they have different features [137].

## 6. Magnetic Components Design Process

**Figure 8:**
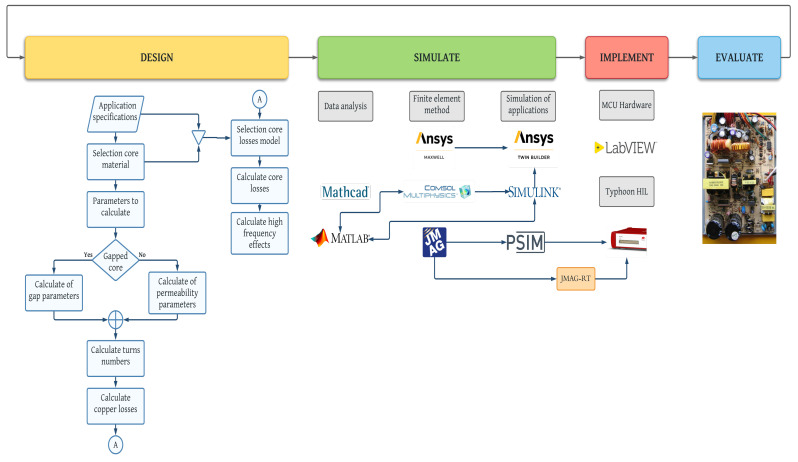
Steps to design a magnetic component. Source: Adapted from [138].

In general, the design process of a magnetic component consists of four steps: design, simulate, implement, and evaluate Figure 8.

The design step can completely follow the diagram shown in Figure 8, where the application specifications will determine the selection core material up to the copper losses, the core losses model, and the high-frequency effects according to [139,140].

It is worth to mention, that there are some intermediate steps, which are related to core physical and magnetic parameters such as the cross section of the core, section of the core, length of it, effective relative permeability, peak to peak density ripple, among others [141]. In the same way, choosing a gapped core (a core with a concentrated air gap) instead of a distributed air gap core will impact its behavior and parameters to calculate; for the first one, gap parameters as length are a priority. For the second one, permeability parameters will be taken into account [142].

As applications specifications as selection core material are fundamental to select or design a core losses model, both will establish the minimum parameters to calculate power losses.

The simulate step is the masterpiece to validate the magnetic component designed, but at the same time, it will be a problematic step if the designer is not careful; FEM software always will give a solution but does not mean that it is correct. In a general way, simulation steps can be subdivided into three parts: data analysis, finite element method, and simulation of applications.

From the design steps depending on the models selected to calculate power losses, core physical parameters, and data related to the windings, many formulas are involved, and sometimes some depend on others; software such as Mathcad^®^ and Matlab^®^ are of great help in these cases. FEM is a big world, as it was explained in the section before. Once the magnetic component’s design is complete (windings and core parameters) and validated in FEM; the next step consists in exporting the final FEM design to software like Simulink^®^ or Twin Builder^®^, PSIM^®^, to simulate it in a specific application and corroborate its correct performance [59,143,144,145,146].

The implementation step tests the simulated application through the interconnection of different sorts of software and platforms (MCU hardware, LabVIEW^®^, Typhoon HIL, among others) to simulate it with high fidelity, in similar conditions to real [147]. Typhoon HIL is a feasible example of this, as it can see in Figure 8. The reader interested in magnetic component testing is referred to [148,149,150,151,152,153] and the references therein.

The last one is to evaluate, that is, physically build the magnetic component designed and tested in the steps before and implement it in a circuit. Technically speaking, the tests obtained in the implementation step must coincide with the physical circuit measurements with a minimal error percentage. If the magnetic component behavior is not satisfactory, the steps must be repeated, modifying the necessary parameters until the desired results are obtained.

## 7. Magnetic Devices and Miniaturization

Transportation, electrification, medical applications, micromachines, wireless communication and wireless charging are some areas demanding new technology to develop compact and high performance applications; and the manufacturing of low losses magnetic components is key [154].

Magnetic components exhibit design difficulties when power conversion systems require low power levels and high frequencies due to the nonlinear behavior of magnetic materials, thermal limits, and the exponential increase of losses under these conditions.

Core data and core losses models are critical to design magnetic components properly and select the size of magnetic devices. According to core selection material different procedures may be applied [155].

Miniaturization of magnetic components and micromachines are an interesting couple to develop and implement in biomedical devices and applications. In biomedical applications magnetism-based systems are widely utilized, for example, to study and measure human tissue and organs (such as magnetocardiography, magnetoneurography, magnetoencephalography, and magnetomyography) [156,157,158,159]. By 2021, the market of magnetic sensors will worth around $7.6 billion due to their attractive commercial purposes and new application areas [160]. However, the development of efficient magnetic technologies that are sensitive, inexpensive, biocompatible and miniaturized is still far away [161].

The miniaturization, feasibility and integration of magnetic sensors for biomagnetic signal detection have been in constant evolution in terms of size, sensing signals magnitude (pico-Tesla scale), and environment conditions. In magnetic sensors, the magnetic field is converted into measurable quantities as voltage and current [156]. Some examples of them are the thin-film magnetoelectric (ME) sensors, optically pumped magnetometers (OPM), superconducting quantum interference device (SQUID), flux gate sensors, giant magnetoimpedance (GMI) sensor, magnetic sensors based on the thin-film magnetoresistive (MR), and conventional superconducting coils. Those are compared in Table 2 [156,160].

In [162], a GMI miniaturized magnetic sensor fabricated with a Co-based amorphous by Micro-Electro-Mechanical System (MEMS) technology wire is described, achieving a size of 5.6 × 1.5 × 1.1 mm${}^{3}$. Co-based amorphous wire is selected for its high impedance change rate, increasing the sensitive of the GMI sensor, it is used to fabricated a pick-up coil of 200 turns and diameter of 200 μm.

A common problem in biomagnetic sensors is the noise at low frequencies, specifically between 10 and 100 Hz due to the small magnitude of measured signals. A set of bi-planar electromagnetic coils is a recent technique to cancelling the Earth’s noise nearby the magnetic sensors and improving their sensitivity [156].

Magnetic signal detection including portable and handled devices in the point-of-care testing (POCT) including mini magnetic induction coils and electromagnets to do the devices more portable, flexible and with high detection capabilities. In [163] several examples of these applications are described, where magnetic devices have dimensions in centimeters scale and those consider other POCT techniques for their favorable accuracy, high reliability, innovation and novelty, although their cost might increase.

Portable and small size, or even single handled devices that provide rapid and accurate detection have potential application of POCT [163]. Portable and wearable biosensors are the future of healthcare sensor technologies. Those have demonstrated their utility in disease diagnosis with accurate prediction. Their incorporation with mobile phones is known as digital health or mobile health, which promises reduce the frequency of clinical visits, prevent health problems and revolutionary the demand of micro and wearable sensors technology [164,165,166].

The emerge of microrobots and the use of a magnetic resonance imaging help to improve diagnostic capabilities by minimally invasive procedures. A microrobot is a robot on a microscale that can perform high-precision operations. Microrobots’ actuation system require input energy to act it, which usually requires special materials (soft and hard magnetic materials) or structural design (arrangement of coils) [167,168]. While the propulsion of microrobots, ferromagnetic cores and magnetic structures can be used to generate an image of many sites in the human body [169,170,171].

In [172], microcoils with a diameter < 1 mm are used to harvest electromagnetic energy wirelessly by inductive coupling in the on-board energy robots, achieving several milliwatts of power, capable of controlled motion and actuation with a maximum efficiency of 40%. This has been a recent development to controlled and powered remotely a system-engineered miniaturized robot (SEMER).

To increase the adaptability and robustness of robotic systems in challenging environments, and inter disciplinary research is needed, including material science, biology, control, among others [172]. Magnetic materials and magnetoelectric concepts of micro and nanorobots in magnetic applications are detailed in [173].

In this authors opinion the versatility, scalability, and flexibility of developing a power core loss model to analyze and allowing a better comprehension of the magnetic phenomena in ferromagnetic materials, will be the first step to the miniaturization of magnetic devices and power conversion systems at any scale [174,175].

## 8. Discussion

**Figure 9:**
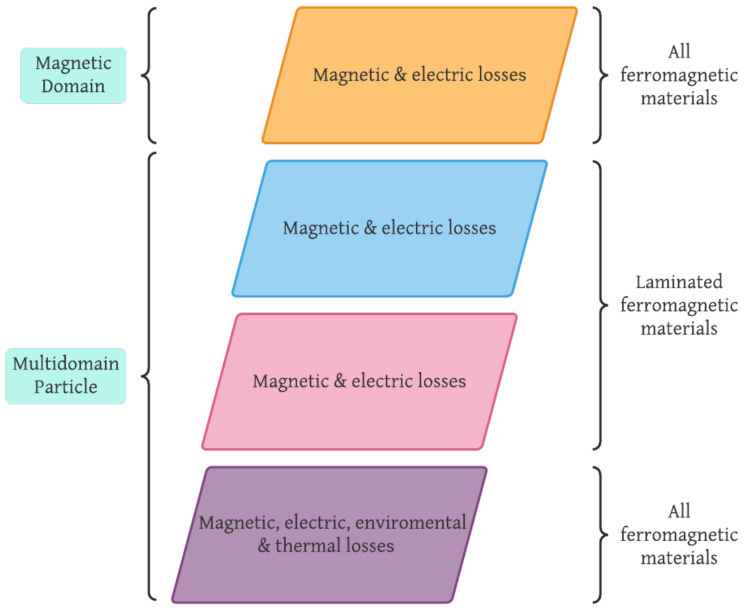
Power core losses models’ comparison depending on the size of the phenomena analyzed, kind of losses to calculate and ferromagnetic materials. Features of mathematical models, time-domain approximations, loss separation, and empirical methods are in color yellow, blue, pink, and purple, respectively.

Nowadays, there are several models for studying, predicting, and analyzing the power losses in the ferromagnetic cores of magnetic components. Usually, these models consider a series of parameters based on magnetic units and frequency to predict and calculate power losses. Figure 9 illustrates a comparison between core losses models depending several features.

Nevertheless, one main application of magnetic components is in power electronics, where the measurement units are electric, which in many cases cause inexactness while designing a magnetic element. A common idea in the literature is that in a magnetic component with high operating frequency, its losses and size will be lower; however, this is not always true. There are many factors involved (kind of material, core losses, copper losses, among others) to determine the integrity of this expression. Although the last years’ power electronic researchers have been working hard to find and develop numerous techniques to achieve better performance from magnetic components, the way is still long. For instance, the future design of magnetic components should take advantage of all capabilities of the final application (support multiple input/outputs, multiple voltage/current domains, characterization of the material, losses’ reduction, among others), with the option to work in parallel with switches at high frequency [12,176].

On the other hand, the industry of FEM software is more versatile each year; several applications and tools are added to these, improving accuracy, functionality, scenarios to apply it, and user friendly environments. Nonetheless, a common point is a lack of compatibility among those, limiting the use of platforms to validate and complement the final design. This lack of compatibility motivates users to know different packages to solve a unique problem.

This is translated into a monetary cost to purchase different licenses and increase computer resources. Therefore, to motivate and improve the FEM designs, in this authors opinion, a standard format type should be implemented in the following years between those kinds of software to take advantage of each characteristic and unify all FEM files.

In the literature, static or dynamic core losses models can be found, depending on the designer choice, the loses calculus could be as complex as the model chosen [177]. So, the model selection in function of the material and the requirements’ application of a magnetic component’s design process are fundamental to achieving an accurate core losses calculus.

Table 3 illustrates a comparison between power core losses models, ferromagnetic materials, and accuracy. It is essential to mention that ferrite is the start point to validate any power core losses model, so this is not listed. Nonetheless, to the best authors knowledge, not all models had been tested in all ferromagnetic materials, or those are not suitable to calculate their power core losses.

Designers can use the information mentioned in Table 3 and Figure 9 to improve some of the power core loss models listed before or propose their model, always keeping in mind that any design parameter will be sacrificed to improve another one; magnetic components size will be reduced with frequency increment.

Due to the high energy consumption worldwide, renewable energy sources and energy efficiency have accelerated research in energy applications, conversion, transportation, and telecommunication [178]. Magnetic materials are the key piece of those research fields, mainly for their potential for energy efficiency and their impact on consumption power [179]. Materials’ demand as electric steel, iron, and cobalt will be exponential growth for 2026. Studies on the effect of magnetic materials in renewable energy have to be a priority to guarantee the supply chain, options for recycling, the footprint impact, the blueprint impact, and the socio-economical impact of their extraction [180,181].

## 9. Conclusions

In the literature, many core-losses models are available to select. Researchers continue developing new core losses models with specific features (core material, waveform, density flux, application, frequency range, among others), but the majority are not easy to replicate.

To develop a power core losses model that involves electrical, magnetic, thermal effects, suitable for all kinds of ferromagnetic materials, and also competent to power electronic is fundamental. Not only for advantages to calculate power core losses accurately but not to reach optimal magnetic components. The challenge is to understand the comportment of the magnetic materials and model them differently than ferrite magnetic elements because, until now, both are very similar, although their behaviour is quite different.

Each of the core losses models in this document is a point of start for researchers. Despite promising results using Steinmetz’s equations, those are not enough to calculate the core losses accuracy according to the need for the power electronic community. FEM software is indispensable nowadays to validate the power losses model as the magnetic component design. At the same time, virtual laboratories are promising to be the trend in a few years, allowing a reduction of the cost of a physical test bench to that of a virtual one and getting good approximations of the magnetic component behavior in a real environment. In addition, the option to interconnect different kinds of software to implement complete power electronics systems in platforms such as HIL, SIL, and MIL, makes the capability of design more versatile.

## Figures and Tables

**Table 1:** Details of Steinmetz’s equations.

Steinmetz’s Equation	Characteristics
OSE	Only for sinusoidal signals.
	Hysteresis losses proportional to *f*.
	Eddy current losses proportional to $f^{2}$.
	The values for α and β are between 1 and 3.
	Equivalent f calculus.
MSE	Considers $B_{s}$ in the core losses.
	Its accuracy decreases with harmonics increment.
	Considers $B_{s}$ variation and its instantaneous value.
GSE	Compensates the mathematical error between OSE and GSE for sinusoidal excitations.
	Considers the DC-level in the signal.
	Takes the peak to peak value of $B_{s}$.
*i*GSE	Accurate with a high number of harmonics.
	Core losses calculus with frequencies and duty cycles variables.
	It can be used for rectangular signals.
NSE	The second and third harmonic are dominants at moderate values of *D*.
	For extreme values of *D* (∼95%) a high alpha value will give a better adjustment.
*i*${}^{2}$GSE	Characteristics similar to *i*GSE.
	Applications with trapezoidal $B_{s}$.
	Takes into account the material relaxation effect.
	Require parameters not provided by the materials manufacturers.
WcSE	Proposed to correlate a not sinusoidal signal with a sinusoidal with the same measured
	value of $B_{s}$.

**Table 2:** Magnetic sensing technologies comparison in biomedical applications.

Magnetic Sensing Technology	Sensitivity	Frequency	Miniaturization Achieving	Portability	Cost
OPM	Acceptable	0 Hz	Unacceptable	Unacceptable	Marginally
					Acceptable
Coils	Acceptable	60 Hz	Unacceptable	Acceptable	Excellent
ME	Acceptable	0–1 kHz	Acceptable	Excellent	Excellent
Fluxgate	Acceptable	0–5 kHz	Excellent	Excellent	Acceptable
GMI	Marginally	0–10 kHz	Excellent	Excellent	Excellent
	Acceptable				
SQUID	Excellent	0–100 kHz	Unacceptable	Unacceptable	Unacceptable
MR	Acceptable	0–GHz	Excellent	Excellent	Excellent

**Table 3:** Comparison between empiric core losses models. The sinusoidal and non-sinusoidal waveform are indicated with light yellow and light green, respectively.

Loss Model	Steinmetz’s Parameters	Additional Data	Ferromagnetic Materials	Characteristics	Accuracy
Preisach	No	Yes	All unless powder cores	Based on domains movements and *B*-*H* loop Many additional data.	Computing cost, approximations
J-A	No	Yes	All unless powder cores	Non-linear equations systems. Based on magnetization core process. Many additional data.	Computing cost, approximations
LSM	No	Yes	All unless powder core	Suitable only for lineal systems. Very accuracy for laminar materials.	Good
TDA	No	No	All unless powder cores	Suitable only for lineal systems. Valid for PWM signals <400 Hz.	Good
OSE	Yes	No	All	Base of empiric loss models.	Low
MSE	Yes	No	All	Not suitable for signals with many harmonics.	$M^{\alpha -2}$
GSE	Yes	No	All	Consider CD-level in the signal.	<*i*GSE
*i*GSE	Yes	No	All	Signals with strong harmonics.	Same as *i*${}^{2}$GSE
NSE	Yes	No	No reported	A similar version of GSE.	No reported
*i*${}^{2}$GSE	Yes	Yes	All	Consider the relaxation effect. Signals with strong harmonics.	Same as *i*GSE
WcSE	Yes	Yes	Nanocrystal, amorphous and powder core	Physical base not verified. A practical and direct method.	D < 0.5
CHW	No	Yes	Nanocrystal, amorphous and powder core	Square waveform as the sum of its components.	Good
Villar	Yes	No	Amorphous material	Core loss calculus implemented a piecewise linear model (PWL).	Good
Górecki	Yes	No	Nanocrystal, amorphous and powder core	Considers thermal, electrical and magnetic effects	Same as manufacturers

## Abbreviations

The following abbreviations are used in this manuscript:

$P_{c}$	Core loss	$P_{h}$	Hysteresis or static loss
$P_{eddy}$	Eddy current loss	$P_{anom}$	Anomalous or residual loss
$k_{h}$	Hysteresis coefficient	$k_{eddy}$	Eddy currents coefficient
$k_{anom}$	Anomalous coefficient	*f*	Fundamental frequency
*B*	Magnetic flux density	$\phi_{n}$	Angle between $B_{n}$ and $H_{n}$
$B_{n}$	Magnetic flux density of the *n*th harmonic	$H_{n}$	Magnetic field of the *n*th harmonic
$B_{s}$	Magnetic induction peak	*x*	Steinmetz coefficient
*d*	Lamination thickness	ρ	Electrical resistivity
*A*	Cross-sectional area lamination	$f_{eq}$	Equivalent frequency
$M_{an}$	Anhysteretic magnetization	$\alpha_{ic}$	Inter-domain coupling
ΔB	Magnetic induction peak to peak	dB(t)/dt	Core loss magnetization rate
β	Magnetic induction exponent	α	Frequency exponent
δ	Magnetizing field direction	$f_{r}$	Fundamental frequency
θ	Sinusoidal waveform phase angle	*T*	Waveform period
*D*	Duty ratio	*N*	Number of turns
FWC	Waveform coefficient	$V_{1}$, $V_{2}$	Voltage
*t*	Time	ω	Zero-voltage period duration
*U*	DC constant voltage	$T_{\omega }$	Length of zero-voltage period
$T_{M}$	Core temperature with smallest losses	$T_{R}$	Core temperature
*M*	Total magnetization	*H*	Magnetic field
$H_{eff}$	Effective magnetic field	$\mu_{0}$	Free-space permeability
$M_{irr}$	Irreversible magnetization	$M_{rev}$	Reversible magnetization
$A_{eff}$	Effective core area	$\alpha_{p}$	Losses temperature coefficient
B(t)	Instantaneous value of core loss magnetization rate	$V_{0}$	Magnetic objects statistical distribution
*G*	Eddy current dimensionless coefficient	$a_{s}$	Anhysteretic magnetization shape parameter
M(t)	Instantaneous magnetization	$M_{s}$	Saturation magnetization
$m_{s}$	Magnetic moment	H(t)	Instantaneous magnetic field
$P_{sqr}$	Rectangular waveform power losses	*c*	Reversibility coefficient
$k_{r}$, $\alpha_{r}$, $\beta_{r}$, τ, $q_{r}$			
*a*, $a_{1}$,$a_{2}$, $f_{0}$, $f_{1}$,	Material parameters	
$f_{2}$, $f_{3}$, $\alpha_{T}$, *k*			
